# A Survey of Rain Attenuation Prediction Models for Terrestrial Links—Current Research Challenges and State-of-the-Art

**DOI:** 10.3390/s21041207

**Published:** 2021-02-09

**Authors:** Md Abdus Samad, Feyisa Debo Diba, Dong-You Choi

**Affiliations:** 1 Department of Information and Communication Engineering, Chosun University, Gwangju 61452, Korea; masamad@chosun.kr (M.A.S.); feyisa2006@yahoo.com (F.D.D.); 2 Department of Electronics and Telecommunication Engineering, International Islamic University Chittagong, Chittagong 4318, Bangladesh; 3 Department of Electronics and Communication Engineering, Adama Science and Technology University, Adama 1888, Ethiopia

**Keywords:** ITU-R model, rain attenuation, millimeter-wave, rain attenuation time series, enhanced synthetic storm technique

## Abstract

Millimeter-wave (30–300 GHz) frequency is a promising candidate for 5G and beyond wireless networks, but atmospheric elements limit radio links at this frequency band. Rainfall is the significant atmospheric element that causes attenuation in the propagated wave, which needs to estimate for the proper operation of fade mitigation technique (FMT). Many models have been proposed in the literature to estimate rain attenuation. Various models have a distinct set of input parameters along with separate estimation mechanisms. This survey has garnered multiple techniques that can generate input dataset for the rain attenuation models. This study extensively investigates the existing terrestrial rain attenuation models. There is no survey of terrestrial rain mitigation models to the best of our knowledge. In this article, the requirements of this survey are first discussed, with various dataset developing techniques. The terrestrial links models are classified, and subsequently, qualitative and quantitative analyses among these terrestrial rain attenuation models are tabulated. Also, a set of error performance evaluation techniques is introduced. Moreover, there is a discussion of open research problems and challenges, especially the exigency for developing a rain attenuation model for the short-ranged link in the *E*-band for 5G and beyond networks.

## 1. Introduction

The rapidly growing demands in high bandwidth, data rate, and availability requirements are pushing to deploy millimeter-wave frequency in wireless networks that can meet these requirements. The reason for such interests is the lower frequency bands’ crowding and increasing demand for high data rates and bandwidth to accommodate ever-increasing customer services [1]. However, the higher frequencies are considerably affected by rainfall. Rain is a natural process that attenuates the propagating signal at microwave and millimeter-wave frequencies. Therefore, it is necessary to mitigate rain attenuation to ensure the quality of microwave and millimeter-wave links. To this end, dynamic attenuation mitigation methods are implemented alongside attenuation prediction models that can predict the projected attenuation of the links. Therefore, multiple studies have been conducted on this issue worldwide. Studies on rain attenuation are used in geographically distributed locations to analyze and develop a rain attenuation model applicable over a wide frequency range, particularly radio frequencies over approximately 30 GHz for 5G and beyond network applications. However, to develop such a rain attenuation model, it is first necessary to determine the factors that affect attenuation. There is evidence that in addition to the rainfall, the frequency, path length (distance between the receiving and transmitting antennas), temperature, wind direction and velocity, pressure, and humidity can affect attenuation. Among others, rainfall intensity, frequency of operation, and link distance are significant parameters that determine rain attenuation. Various rain attenuation prediction models have mapped the correlation between rainfall intensity, path length, and frequencies with rain attenuation. An increment in rainfall rate increases the chance of interfering probability with radio waves [2]. In some studies, attenuation because of rain was reported at even lower frequencies, such as 5 GHz [3] and 7 GHz [4]. Many rain attenuation models have been proposed in the literature, and researchers have attempted to improve existing models to fit with local climatic conditions [5,6,7,8,9,10,11,12]. The accurate estimation of attenuation because of rain in a specific radio link is essential for planning the link budget, maintaining the link quality, and designing the system. It was shown that rain attenuation could reduce the throughput of a link compared to sunny weather conditions [13]. By deploying an appropriate rain attenuation model, even in the rain, a terrestrial link’s throughput can be kept unchanged compared to a case without deploying any FMT and with the condition that other parts are usually working. FMT might be attained in several ways, such as power control, modulation techniques, adaptive waveform, and diversity techniques. If we do not consider power control of FMT owing to rain attenuation, then it is not possible to avoid overestimating or underestimating a transmission system’s power. In fact, in every frequency band, the radio frequency (RF) engineer must follow the allowable power transmission requirements according to the spectrum management regulatory organization’s rule. Thus overestimated the power of the transmitted signal strength may create interference into another frequency spectrum that is in use in the neighboring device if the engineers do not adhere to such specifications. On the other hand, underestimated power in the transmitted signal may further be attenuated by rain attenuation if the effect of rain is not mitigated either by changing modulation technique, adaptive waveform, or diversity control. Thus rain attenuation model plays a significant role in the FMT operation in a transmission system. As said earlier, higher rate data transmission demand leads to implement and use 5G and beyond wireless networks where millimeter-wave frequency band is a powerful candidate. However, the millimeter-wave coverage is short, leading to a higher number of terrestrial links in the 5G and beyond network. Thus, for the 5G and beyond the network’s proper operation, accurate rain attenuation model deployment is substantial for terrestrial link. In this regard, lots of terrestrial rain attenuation models are proposed. However, a survey paper that contains an in-depth analysis of the models is not available, to the best of found knowledge. We have tried to fill this gap through this study. This paper exhibits parameters affecting rain attenuation, classification of recognized models, microwave and millimeter-wave frequency bands applicability to terrestrial links, efficient path length determination techniques, and enhancements or shortcomings of the models. Besides, a critical review of 18 well-known rain attenuation prediction models is assessed, classified, evaluated, compared, and summarized in this study. It is mentionable that previously we studied thoroughly brand-new learning-based rain attenuation models in [14]. That paper substantially differs from the current study as the main concentration of that article was learning-based rain attenuation models, including terrestrial and slant links. In the remainder of this study, the term model refers to the rain attenuation model and ITU-R (International Telecommunication Union- Radio-communication sector) database or DBSG3 (Study Group 3 databanks) will be used interchangeably.

### Contributions

This article thoroughly analyzes the main features, weaknesses, and unique characteristics of well-known and new models. In this article, the main points are:

- Section 2 includes extensive coverage on predicting the accurate rain rate for the ungauged area, techniques to generate rain rate corresponding attenuation time series (Table 1), highly spatial resolution rain rate estimation techniques (Table 2), and effective path length through correction techniques (Table 3).
- To the best of our knowledge, there is no survey paper regarding the prediction of the rain attenuation of terrestrial links. We classified the most well-known and updated models in this study, which are presented in Section 3.
- We developed a brief overview of each of the selected models. The quantitative and qualitative features of various models are tabulated in Tables 6 and 7, respectively.
- We observed an inherent improvement in each model, criticizing the model by finding the drawbacks and unique features mentioned in Table 8.
- The comprehensive research concerns are summarized in Section 6.
- We have tabulated recent research works outcomes with short-ranged links at 26, 38, 58, 72, 73, 75, 77.52, 84, and 120 GHz frequency where well-known ITU-R model predicts inaccurately (Table 9).

## 2. Preliminaries

This section discusses rain attenuation factors, data collection, available databases, experimental studies, and database sources to check the validity of the model.

### 2.1. Rain Attenuation Factors

It is crucial to find a justification and insightful analysis to determine the variables that influence rain attenuation. Although rain is a crucial factor influencing rain attenuation, the link distance, frequency, and polarization play a significant role in the determination of rain attenuation. A brief review of more parameters for rain attenuation is presented here. In the literature, various researchers have found different rain attenuation factors for either terrestrial or slant links. In this regard, we compiled 17 parameters that can impact rain attenuation for microwave links using artificial or ML-based techniques [14].

### 2.2. Rainfall Rate Data Collection Procedures

The rain rate is an essential parameter for determining rain attenuation. In this section, different data collection techniques for rain attenuation are discussed.

#### 2.2.1. Available Databases

A newly devised model should check the efficiency for its validity the confirmation. In most cases, the model developer uses the ITU-R DBSG3 rain attenuation database. In some cases, weather databases *European Center for Medium-Range Weather Forecasts* (ECMWF) or *ECMWF re-analysis-15* (ERA-15) were also used as secondary sources of determining the rainfall rate. These secondary databases lack rain attenuation information on terrestrial and earth-space links for tropical regions. Consequently, most of the models developed in tropical countries are needed to create facilities to prepare the rain attenuation databases.

#### 2.2.2. Experimental Setup

A simple method of determining the rain rate is to set up an experiment to deploy measuring equipment such as the use of a disdrometer, weather station, and rain gauges that measure the rain rate at lower integration times (⩽1 min intervals), which can be saved in a personal computer with the help of a dedicated data logger [2,5,15,16]. In some cases, the radar information of the rain cell was used to measure the rain rate. The problem with radar-based techniques is that massive investments are required to collect rain rate information if radar systems have not been deployed for other purposes [17,18].

#### 2.2.3. Rain Rate Data Generation: Synthetic Technique and Logged Data

The rain rate time series in a specific area is essential because it is used to calculate the attenuation in a fixed radio transceiver infrastructure [19]. The general procedure for collecting the rain rate time series is to collect the data by employing an experimental setup. Thus, the general approach is time-consuming because a minimum of one year of data should be collected over a particular area. Cost is also associated with this process. In addition to this experimental technique, a synthetic method can be used to calculate the time series using a mathematical approach. Table 1, summarizes the various types of synthetic time series assessment techniques.

#### 2.2.4. Rain Rate Prediction from Spatial Interpolation Techniques

To accurately determine the rain attenuation, it is necessary to consider the spatial distribution of the rainfall intensity. The rain rate cannot be measured everywhere using the rain rate collector, which significantly reduces the accuracy of the experimental setup. However, an intense spatial resolution rain rate is required for accurate estimation. There exist some synthetic techniques by which the undetermined rain rate can be estimated to solve the problem at a particular location.

The inverse distance weighting (IDW) technique as per Equation (1) can be used to determine the rainfall rate at ungauged locations [20,21]:

(1) $$R_{p}=\sum_{i=1}^{N}w_{i}R_{i},$$

where *N* is the number of rain gauges. The rain value $w_{i}$ depends on the location of $d_{i}$ in the estimated position *p* is given by Equation (1), and $w_{i}$ is given by Equation (2):

(2) $$w_{i}=\frac{d_{i}^{-2}}{\sum_{i=1}^{N}d_{i}^{-2}}.$$

The average rainfall rate was then determined from these estimated values, along with the rain gauge readings used in this analysis. Using Equation (1) the rain rate can be predicted up to 10–30 km. Unfortunately, the rainfall data available in the weather database ERA-40 provided by the ECMWF suffer from a low spatial resolution $1.125^{\circ }\times 1.125^{\circ }$ latitude per longitude grid.

The spatial-temporal rainfall distribution mechanisms based on the top-to-bottom data analysis approaches are surveyed in [22]. This survey compared most techniques that predict high-resolution space-time rainfall using remote sensing, conventional spatial interpolation, atmospheric re-analysis of rainfall, and multi-source blending techniques, and discussed issues in integrating various merging algorithms. In the article, it was shown that the maximum spatial resolution is available by the *Global Satellite Mapping of Precipitation Near Real-Time (GSMaP-NRT)* dataset with a resolution of up to $0.01^{\circ }$ with an update of once per hour, which is clearly higher than the ECMWF database. Table 2 presents an analysis of different high-resolution spatial rainfall estimation techniques.

Another technique for generating the rain rate is applying the local rain data to the MultiEXCELL model [23]. This model was used in [24] to generate synthetic rain rates. Transmitting and detecting specific differential phase-shifted signals through a dual-band radar system has been experimented with in [25]. As a result of this experiment, the authors noticed the scattering effects in the detected signals that arise due to the radar signals’ differential reflection. A corrector factor should be used for the reflected and differently reflected signals in order to eliminate the scattering effects. The statistical uncertainties of rainfall are then calculated by considering the propagation of the power-law relations.

(3) $$R\left(Z_{h},Z_{dr},K_{dp}\right)=9.6046Z_{h}^{0.072}Z_{dr}^{-0.017}K_{dp}^{0.824}.$$

**Table 1:** Estimation techniques of rain attenuation time series.

Ref.	Estimation Techniques
[26]	The proposed technique generates rain attenuation time series using storm speeds from 1 to 12 m/s in a two-layered rain structure model. Also, temperature, altitude, and height are used as per the geographic location.
[27]	$A(t)=\frac{a_{0}\cdot e^{\sqrt{2d_{AG}/\beta_{a}}\cdot W\left(t\right)+d_{AG}\cdot v_{a}/\beta_{a}\cdot t}}{1+d_{AG}\cdot a_{0}\int_{0}^{t}e^{\sqrt{2d_{AG}/\beta_{a}}\cdot W\left(s\right)+d_{AG}\cdot v_{a}/\beta_{a}\cdot s}ds}$ where $a_{0}$:0–0.5 dB, W(t): Wiener process, $\beta_{a}$, $v_{a}$: gamma distribution parameters, $d_{AG}$: Dynamic parameter β of the Maseng-Bakken model.
[28]	It proposed an enhanced technique to generate rain attenuation time series where precise rain rates are not available at global scale using ITU-R model. The technique uses mean and standard deviation of rain rate either from NOAA [29] and ITU-R model [30] and the output of Gaussian noise through a low-pass filter (LPF: k/p+β, cut-off frequency f_c_: 0.2 MHz) into a non-linear memoryless device, where $A_{\mathrm{offset}}$ is the calibration factor, $A_{\mathrm{offset}}:\;exp(m+\sigma Q^{-1}(P_{0}/100))$ and *Q*: zero-mean, unit variance Gaussian probability density function.
[31]	$A\left(x_{0}\right)=k_{A}\int_{0}^{L_{A}}R^{\alpha_{A}}\left(x_{0}+\Delta x_{0},\xi \right)d\xi +k_{B}\int_{L_{A}}^{L_{B}}R^{\alpha_{B}}\left(x_{0},\xi \right)d\xi$ where $L_{A}$ and $L_{B}$ are the radio path lengths, $\Delta x_{0}$ is the shift due to the presence of layer B, $x_{0}=v\cdot t$, and *v* is the average storm speed (typically 10 m/s).
[32]	$A\left(t_{0}\right)=\frac{1}{\cos\theta }\left[\int_{d_{0}}^{d_{0}+S_{A}}k_{A}R{\left(l\right)}^{\alpha_{A}}dl\right.\left.+\int_{d_{0}+S_{A}}^{d_{0}+S_{A}+S_{B}}k_{B}3.134^{\alpha_{B}}R{\left(l\right)}^{\alpha_{B}}\;dl\right]$, where θ:link elevation angle, ($\alpha_{A}$, $k_{A}$), ($\alpha_{B}$, $k_{B}$): power-law coefficients that converts the rain rate into specific attenuation for layers A and B, respectively, and *R*: rain rate along the link.
[33]	A copula is a multivariate distribution function expressed by marginally uniform random unit interval variables and it can avoid dependence index like in log-normal distribution. The procedure is: $\rho =\sin\left(\frac{\pi \tau }{2}\right)\to$ zero mean Gaussian random variables correlated matrix→normal CDF→desired random variable→inverse CDF of the desired distribution.
[34]	The procedure is: $R_{G}=e^{-\beta \cdot |\tau |}\to \left[\beta_{EMB},\beta_{gamma}\right]\to H\left(z\right)=\frac{\sqrt{1-e^{-2\beta T_{s}}}}{1-z^{-1}e^{-\beta T_{s}}}$, where $T_{s}$: sampling time, and $\beta_{EMB}$ and $\beta_{gamma}$ are $12.3d^{-0.95}\times 10^{-4}$ and $6.9d^{-0.6}\times 10^{-4}$, respectively.
[35]	The procedure is: $a_{k}=\frac{1}{N}\sum_{j=0}^{N-1}M_{j}e^{-\frac{i2\pi }{N}kj}=ℑ\left(M_{j}\right)\to a_{k}=\sqrt{h_{k}ℑ\left(c_{G}\right)}\times e_{k}\to M_{j}={ℑ}^{-1}\left(a_{k}\right),\left[h_{k}=0.5\right]$, where M(t): Gaussian process, *ℑ* and ${ℑ}^{-1}$ are direct and inverse Fourier transforms, respectively.
[36]	Compute the stochastic differential equation: $dA\left(t\right)=\frac{\mu }{4}d_{a}\left(\frac{\mu^{2}}{\lambda }A\left(t\right)-A^{2}\left(t\right)+\mu^{2}\right).dt\;+\sqrt{d_{a}\frac{\mu^{3}}{\lambda }}A\left(t\right)dW\left(t\right)$, where $d_{a}=2\beta_{a}S_{a}^{2}\frac{\lambda }{\mu^{3}}$, where μ and γ are found by fitting to experimental first order statistics of rain attenuation, $\beta_{a}$ and $S_{a}$ are the parameters of the diffusion coefficient of the M-B model.
[37]	Compute:$P\left(t_{i}\right)=1-P_{0,i}\to z_{i}=T_{z}\left(r_{i}\right)\to \mathrm{find}\;M_{z}\left(d\right)\to \mathrm{Gaussian}\;\mathrm{PDF}\to \rho_{j}\left(\tau \right)\to H_{i}\left(z\right)$, where $P_{0,i}$ is the possibility of rain in the *i*th station, $r_{i}$ represents a nonlinear transformation $T_{z}$, and $\rho_{j}$ is the temporal autocorrelation function of rain attenuation for *i*th link.

**Table 2:** Highly spatial resolution rainfall estimation models.

Ref.	Technique or Resolution
[5]	Analyzed millimeter-wave and showed that the ITU-R predicted rainfall rate of region P is up to 0.01% of time (agrees → 99.99% of time and disagrees → 0.01% of time).
[22]	This multi-source blending technique to estimate high-resolution space-time rainfall scales to develop and merge remote sensing, conventional spatial interpolation, atmospheric re-analysis of rainfall, and multi-source blending techniques.
[38]	It presented gauged-based data re-analysis at a resolution of $0.5{}^{\circ }\times 0.5{}^{\circ }$.
[39]	In GSMaP-NRT, it analyzed the satellite, microwave-infrared, and near real time weather dataset to compare better predictability presented resolution about $0.01{}^{\circ }\times 0.01{}^{\circ }$.
[40]	ECMWF: $1.125{}^{\circ }\times 1.125{}^{\circ }$
[41]	It proposed the spatial and the temporal correlation functions to determine rainfall rate.

**Table 3:** Techniques to calculate effective path length (EPL) or path length coefficient factor (PCF).

Ref.	EPL or PCF	Parameter Settings	Remarks
[2]	$r=1/\left\{1+0.03{\left(100P\right)}^{\beta l^{m}}\right\}$ ${}^{⧫}$	Method: Practical measurement; Frequency band: 7–38 GHz; Path length: 58 km; and rain rates were collected over 1-min time interval.	The correction factor depends on β; link length; and p% of rain
[3]	$r_{rad}\left(t\right)={A_{rad,}}_{d}\left(t\right)/\gamma_{R}d$	Method: Simulation Frequency: 22 and 38 GHz Path lengths: 2, 5, 10 and 20 km	The correction factor only depends on the Arad,d(t), and γR(t).
[42]	$r=\frac{1}{1+\frac{L}{\frac{2636}{R(P)-6.2}}}$	Method: Practical measurement Rain rate: 5-min point at 11 GHz frequency; 42.5 km long radio link with R>10mm/h	The correction factor depends on the radio link length and rain rate.
[43]	$r=1.08L^{-0.5108}$ (7 GHz for 0.01% of the time)	Method: Practical virtual link; Link length: 1–10 km; Time exceedance: 0.01%; Frequency 7 GHz	The reduction function depends only on the total path length. Estimation: Exponential curve fitting
[44]	$d_{eff}=\frac{1}{1+d/d_{0}}\cdot d$	Method: Practical setup; Site: S. Paulo, Brazil; Season: Dry season; Frequency: 15 GHz (4 links) and 18 GHz (2 links) with vertical and horizontal polarizations; Path lengths: 7.5–43 km; Duration: 1–2 year	The correction factor depends only on the rain rate exceedance of p% of the time. Estimation: exponential curve fitting
[45]	$r\left(R_{0.01},L\right)=L\times \left(\frac{-R_{0.01}}{1+\zeta \left(L\right)\times R_{0.01}}\right)$ ${}^{▴}$	ITU-R database; Site: 8 countries; Path lengths: 1.3–58 km; Frequency: 11.5–39 GHz; Rain rates (0.1%): 18–105mm/h	The PCF depends on rain rate exceedance %p of time and link length. Estimation: curve fitting
[46]	$r=3.6435{R_{p}}^{-0.377}$	Method: Practical setup; Link length: 2.29 km; Rain Gauge: Tipping rain bucket (0.254 mm accuracy); Frequency: 28.75 GHz	The correction factor depends only on the rain rate exceedance of the %p of the time. Estimation: curve fitting.
[47]	$r\left(R_{0.01},d\right)=d/\left[1+\left\{d/2.6379{R_{0.01}}^{0.21}\;\right\}\right]\;$	Practical setup; Link length: unavailable; Rain Gauge: Tipping, Frequency: 15 GHz, Availability: 99.95%; Duration: 4 years; Rain rate: R (0.1 to 0.001)	The correction factor depends only on the rain rate exceedance of the %p of the time and LOS link length. Estimation: exponential curve fitting
[48]	$r=1.303\frac{\varsigma }{1+\frac{L}{D}}$ ${}^{♣}$	Model: empirical model, based on the point of inflexion (POI)	The correction factor depends only on the slant path length and the rain cell diameter.
[49]	$r=A/\left(kR_{TX}^{\alpha }L_{slant}\right)$	Method: MultiEXCELL rain simulation. Calculation: rain attenuation is calculated via the numerical approach. Rain field size: 1 km × 1 km to 250 km × 250 km	The correction factor depends on calculated attenuation, specific attenuation conversion coefficients, ‘measured’ rain rate at the transmitter end, and the LOS link length.
[50]	$r=\frac{1}{\left[0.477L^{0.633}R_{0.01\%}^{0.073\alpha }f^{0.123}-10.579\left(1-e^{-0.024L}\right)\right]}$	(1) Can be used worldwide; (2) frequency band: 5–100 GHz; (3) Maximum path length is 60 km	The correction factor depends on the frequency (GHz), specific attenuation coefficient (α), and link length (L).
[51]	$r=\left\{\begin{array}{cc} \frac{N_{e}}{\cos\theta }\left(\frac{h_{R}-h_{S}}{\tan\theta }\right) & R<R_{0}\\ \frac{N_{e}}{\cos\theta }\left(\frac{1}{0.056+0.012R}\right) & R\geq R_{0} \end{array}\right.$	The number of effective cells $\left(N_{e}\right)$ is calculated after analyzing ITU-R DBSG3 database.	To define the rain cell, it needs to know the cell $\left(R_{0}\right)$ boundary rain rate.
[52]	$r=\left\{\begin{array}{cc} \left[\frac{1}{1.77d^{0.77}R_{0.01}^{-0.05}}\right] & f\leq 40\;\mathrm{GHz}\\ {\left[\frac{1}{0.477d^{0.633}R_{0.01}^{0.073}f^{0.123}}\right]}^{2} & f>40\;\mathrm{GHz} \end{array}\right.$	It was based on the measured attenuation of smaller than 1 km terrestrial link and frequency 26/38 GHz.	It concluded that the distance factor is inconsistent for a link length smaller than 1 km.

⧫ m(F,l)=1+Ψ(F)lnl and $\Psi (F)=1.4\times 10^{-4}F^{1.76}$ the value of depends on path length and the considered rain percentage exceedance, ${}^{▴}\;\zeta (L)=-100$ when L≤7km and $\zeta (L)={\left\lceil \left(\frac{44.2}{L}\right)\right\rceil }^{0.78}$ when L>7km, ${}^{♣}\;\zeta_{0.01}=\zeta_{1}$; $R_{0.01}\geq 110$mm/h, otherwise $\zeta_{0.01}=S_{2}$ or $\zeta_{2}S_{3}$

**Table 4:** Error estimation techniques for rain rate prediction

Ref.	Technique or Resolution	Remarks
[53]	$E=\frac{1}{nCountry}\sum_{i=1}^{415}W_{i}\left|\log\left(\frac{{R_{i}}^{DBSG3}}{{R_{i}}^{S-B}}\right)\right|$	This test was used to *re-analysis* based rain rate and the rain rate provided by the ITU-R DBSG3 database.
[54]	$E_{R0.01}=\sqrt{E_{\psi }^{2}+E_{\phi }^{2}+{\Delta R_{0.01}}^{2}}$ where $E_{\psi }^{2}={\left(\frac{\partial R_{0.01}}{\partial \psi }\right)}^{2}\sigma_{\psi }^{2}$ and $E_{\phi }^{2}={\left(\frac{\partial R_{0.01}}{\partial \phi }\right)}^{2}\sigma_{\phi }^{2}$.	The model was developed and verified using DBGS3 along with CHIRPS rainfall (ψ), and TPW (ϕ) in the ERA-Interim Reanalysis database. Authors have not compared with measured data and the precise calculation of rain rate $R_{0.01}$ showed lower accuracy (uncertainty is about 14%).

The wet-antenna effect has relation with the bias value of the signal in the receiver section. However, an appropriate bias compensation technique has not yet been developed.

A rain-rate-retrieval algorithm was designed using radar reflectivity derived from the rain rate in [55]. Based on the Doppler velocity, the derived radar reflectivity was classified as low-and high-rain cases. This model paved the way for blending reflectivity and attenuation to predict the rain rate. However, beyond reflectivity and attenuation, other factors, such as seasonal variation and rain type, were not considered.

The minimum observed attenuation and the maximum observed attenuation were calculated through a *commercial microwave link (CML)* within a fixed interval [56]. Using these minima and maxima, the observed attenuation value averaged rain-intensity can be calculated as

(4) $${\bar{R}}_{i}=\sqrt[{b}]{\left(\frac{\max(A_{i}^{r\_\max}-B,0)}{\tilde{\alpha }L}\right)},$$

where B (in dB) is the induced bias value because of to the mixture of the transformation of the min/max with the quantizer, the negative values of ($A_{i}^{r\_\max}-B$) are counted as zeroes when they exist, $\tilde{a}=a\cdot {\left[\ln\left(K\right)+0.57722\right]}^{b}$, and the *a* and *b* parameters refer to the power-law relationship of specific coefficients and *K* is the number of instantaneous samples per interval from which the maximum attenuation is extracted.

Since 2000, numerical weather prediction (NWP) has become popular in predicting rainfall and has drawn interest from the meteorological forecasting industries, researchers, and other stakeholders. However, owing to decreased portability and implementation coverage in remote locations, NWP-based techniques are not a potential technique for remote area application. Therefore, the prediction of learning supported rain diminution is standard because the problem of the NWP technique can be solved. In [57,58,59,60,61,62,63] ML-based rainfall prediction techniques were presented. Table 4 lists some of the error estimation techniques for rain rate prediction.

### 2.3. Distance Correction

The rain attenuation (*A*) was calculated by multiplying the specific attenuation and the distance between the transmitting and receiving antennas.

(5) $$A=\gamma L_{e}.$$

Assuming the effective path length $L_{e}$ to be 1 km, in Equation (5) the specific attenuation and the link attenuation are equal. Equation (5) is true if the rain and cloud are uniformly distributed over the entire path between the transmitting and receiving antennas. However, if the distance between the transmitting and receiving antennas is not 1 km:

(6) $$L_{e}=\frac{A}{\gamma }.$$

Owing to the non-uniform distribution of rain, the values of the specific and link attenuation (for 1 km length) are different, which defines a term called the effective path length. This implies that the effectual and actual distance varies for non-uniform rain distributions and links. The effectual distance is usually calculated based on the rainfall distribution [2]. Many models calculate the effective path length using a correction factor, referred to as the path adjustment factor. In terrestrial links, all the link lengths remain within a single rain cell for a short link or many cells for a long link. A brief discussion on the parameters that affect either the effective path length or path length adjustment factor is presented in the next section. In most cases, the accuracy of the model discussed above was calculated using the measured rain attenuation data, which was then compared to the attenuation derived through the attenuation formula. In some cases, the root means square (RMS) and standard deviation (STD) values were calculated to validate the model. Table 3 contains all of the most critical effective path length or distance correction factors proposed in the literature. The distance correction factor is more crucial for the *E*-band, which is a probable frequency band for developing 5G and beyond wireless communication networks, as discussed in Section 6.4.

### 2.4. Frequency and Polarization

The specific attenuation can be determined from the rainfall rate, frequency and polarization using the following power-law relation [30,64,65].

(7) $$A_{sp}\left(dB/km\right)=xR_{0.01}^{y},$$

where $R_{0.01}$ is the rain rate, and x and y are regression coefficients that depend on several factors such as: polarization, carrier frequency, temperature, and rain drop size distribution [64]. The values of x and y can be determined experimentally as empirical values. ITU-R P. 838-3 [30] provides the prediction values for x and y for 1–100 GHz frequency bands at horizontal and vertical polarizations.

In this section, various parameters of rain attenuation, rain rate data collection procedure, available public domain databases, time-series generation techniques, percentage of time exceedance of rain (Equation (7)), specific attenuation coefficient determination procedure, and the procedure of distance correction factor have been discussed. All the data collected or modified through these techniques can be used by the rain attenuation models, which will be discussed in the next section.

## 3. Rain Attenuation Models: Terrestrial Links

Existing terrestrial models can be classified into five categories based on the formulation of the rain attenuation model. These include the empirical, physical, statistical, fade slope, and optimization-based models.

- **Empiricalmodel:** The model is based on experimental data observations rather than input-output relationships that can be mathematically described. The model is then classified as an empirical category.
- **Physical model:** The physical model is based on some of the similarities between the rain attenuation model’s formulation and the physical structure of rain events.
- **Statistical model:** This approach is based on statistical weather and infrastructural data analysis, and the final model is built as a result of regression analysis in most cases.
- **Fade slope model:** In the fade slope model, the slope of attenuation from the rain attenuation versus time data was developed with a particular experimental setup. Later, these data were used to predict rain attenuation.
- **Optimization-based model:** In this type of model, the input parameters of some of the other factors that affect the rain attenuation are developed through optimization (e.g., minimum error value) process.

**Figure 1:**
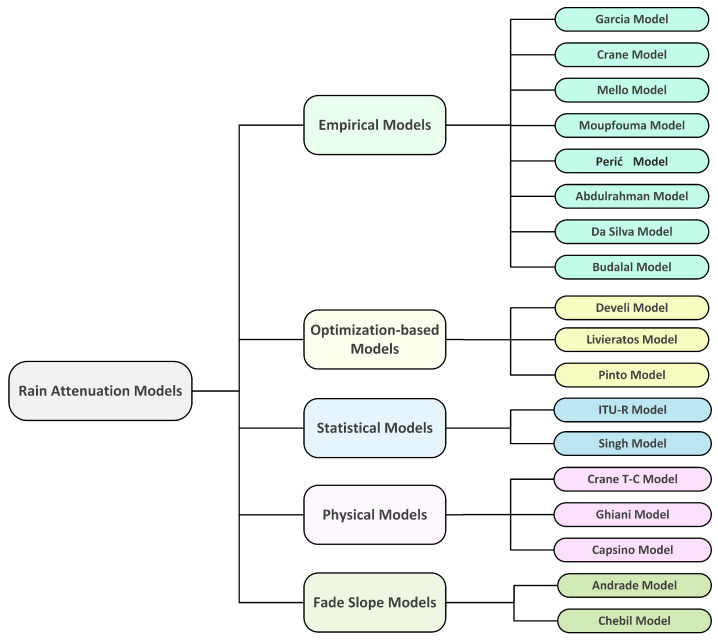
Taxonomy of terrestrial rain attenuation models.

Figure 1, represents a taxonomy of the well-known and recently developed rain attenuation models used in this study.

### 3.1. Empirical Models

#### 3.1.1. Moupfouma Model

This model [45] uses the rain rate exceeded by 0.01 percent of the time and the calculation of the proportion of time-correlated with the excess of any given interest attenuation.

(8) $$\gamma_{R_{0.01}}=kR_{0.01}^{\alpha }$$

(9) $$A_{0.01}=\gamma_{R_{0.01}}\times L_{eq},$$

where $L_{eq}$ is the equivalent path length for which the rain propagation is assumed to be uniform.

#### 3.1.2. Budalal Model

In this model [52] according to the 300 m link’s attenuation analysis with frequencies of 26 and 38 GHz, the authors found attenuation inconsistency provided by the latest ITU-R model. They then investigate the specific attenuation ($\gamma_{th}$) as per ITU-R P.838-3 [30] and found an inconsistency between the effective specific attenuation ($\gamma_{eff}$) can be defined as Equations (10) and (11):

(10) $$I_{f\gamma }=\left[\frac{1}{1.77d^{0.77}\;R_{0.01}^{-0.05}\;}\right],\;\mathrm{for}\;f\leq 40\;\mathrm{GHz},\;d<1\;\mathrm{km}$$

(11) $$I_{f\gamma }\;=\;{\left[\frac{1}{0.477d^{0.633}\;R_{0.01}^{0.073}\;f^{0.123}}\right]}^{2},\;\mathrm{for}\;f\;>\;40\;\mathrm{GHz},\;d<1\;\mathrm{km}.$$

It is inferred that the model can be used for short-range outdoor links with frequencies higher than 25 GHz in 5G networks.

#### 3.1.3. Perić Model

This model is also referred to as a dynamic model [66]. It depends on the cumulative distribution function of the rain intensity of the area of interest, the number of rain events in which the rain intensity threshold is exceeded, the rain advection vector intensity, and the rain advection vector azimuth. The model considers the spatial distribution within a 10 km radius around an antenna and is suitable for small geographical areas, up to 10 km × 10 km. Furthermore, it has not been tested in a real-world network environment.

#### 3.1.4. Garcia Model

It is one of the modified version [67] of Lin model [42], assuming that the *path length reduction coefficient* changes with the path length and rainfall rate. The developed model was tested with in Paris, Stockholm, Dijon (France), and Kjeller (Norway), with variations in frequency and path length. The model is best suited for temperate European regions.

(12) $$A=kR_{1-min}^{\alpha }L\frac{1}{0.5+[L(3R_{1-min}-3.9L+245)/2636\;]},\;\mathrm{for}\;R>\;10\;\mathrm{mm}/h\;,\;L>\;5\;\mathrm{km}.$$

This model improves the limitation of the 5-min rain rate requirement of the original Lin’s model. This model’s drawback is that it was only tested at 11 GHz and not at higher frequencies. Furthermore, the model did not consider spatial rain distribution variations. Another limitation of this model is that it only applies to terrestrial links.

#### 3.1.5. Da Silva/Unified Model

This model [68] uses the full rainfall rate distribution with multiple nonlinear regressions from the rain attenuation database. It is primarily developed for terrestrial links and can be later extended to slant links. For a terrestrial link,

(13) $$A_{p}=k{\left[(R_{effT}\left(R_{p},d\right))\right]}^{\alpha }.\frac{d}{1+d/d_{0}\left(R_{p}\right)\;},$$

where $R_{effT}$ is the approximate effective rain rate for terrestrial links and the and the cell diameter $d_{0}$ is given by Equations (14) and (15) respectively.

(14) $$R_{effT}=1.74R^{0.786+0.197/d\;}$$

(15) $$d_{0}=125R^{-0.33}$$

(16) $$R_{eff}\left(R_{p},L_{s},\theta \right)=1.74R^{0.786+0.197/L_{s}\cos\theta }\cdot \left(\cos\theta +\frac{120}{{L_{s}}^{2.88}}R^{-0.186}\sin\theta \right).$$

For the terrestrial case $L_{s}=d$, the second term in the brackets vanishes as $\theta =0^{\circ }$, and the expression is reduced to the terrestrial case prediction method. With the correct consistency for terrestrial and slant paths, the model exhibits good performance; however, the error has not been compared with real attenuation data.

#### 3.1.6. Mello Model

According to this model [69] the cumulative probability distribution of rain attenuation for terrestrial link can be determined by the Equation (17):

(17) $$A_{p}=k{\left[1.763R^{0.753+0.197/L_{s}\cos\theta }\right]}^{\alpha }\frac{d}{1+\frac{d}{119R^{-0.244}}}.$$

#### 3.1.7. Abdulrahman Model

According to this model [70] the rain attenuation is given by the Equation (18):

(18) A%p=μ[S(R%p)]

where

(19) $$S\left(R\%p\right)=\beta R_{\%p}^{\alpha -1}$$

(20) $$\beta =k\left[\alpha +b\left(1-r\%p\right)\right]d_{\mathrm{eff}}$$

(21) $$\mu =\left[\frac{R\%p}{\alpha +b(1-r\%p)}\right].$$

#### 3.1.8. Crane Model

This model [71] establishes rain distribution from a global perspective and the USA’s precise rain distribution maps. From these maps, the rain rate distribution can be calculated.

If the path length D>22.5km, then the rain rate should be modified:

(22) $$R_{P}^{\prime }=R_{P}\left[\frac{D_{0}}{D}\right]$$

where $D_{0}=22.5\;\mathrm{km}$

(23) $$\begin{array}{c} \begin{array}{c} A\left(R_{p},D\right)=kR_{p}^{\alpha }\left[\frac{e^{u\alpha d}-1}{u\alpha }-\frac{b^{\alpha }e^{c\alpha d}}{c\alpha }+\frac{b^{\alpha }e^{c\alpha D}}{c\alpha }\right],\;d⩽D⩽22.5\;\mathrm{km}\\ A\left(R_{p},D\right)=kR_{p}^{\alpha }\left[\frac{e^{u\alpha D}-1}{u\alpha }\right],\;0<D⩽d \end{array} \end{array}$$

where the constants are given by Equation (24)

(24) $$\begin{array}{c} \begin{array}{c} u=\frac{\ln\left[be^{cd}\right]}{d},\;d\;\mathrm{in}\;\mathrm{km}\\ b=2.3R_{p}^{-0.17},\;R_{p}\;\mathrm{in}\;\mathrm{mm}/h\\ c=0.026-0.03\ln R_{p}\\ d=3.8-0.6\ln R_{p}. \end{array} \end{array}$$

### 3.2. Physical Models

#### 3.2.1. Crane Two-Component (T-C) Model

This model [72] is based on different integration techniques for heavy and light rainfall regions. The author proposed two versions of the T-C models: the first is a simple T-C model and was published in 1982. The model consisted of several steps. (1) Determining the propagation path for the global climate. (2) Finding a mathematical relation between the projected path length in the rain cell and debris region. (3) Fixing the expected amount of attenuation. (4) Deriving the required rain rate to produce rain attenuation and calculating the probability that the specified attenuation is fixed in step (3).

(25) $$P\left(a>A\right)=P_{c}\left(a+D_{c}/W_{c}\;\right)e^{-R^{\prime }/R_{c}\;}+P_{D}\left(1+D_{D}/{W^{n}}_{D}\;\right)\eta \left(\frac{\ln R^{n}-\ln R_{D}}{\sigma_{D}}\right).$$

The model was primarily developed for Western Europe and the USA, and has difficulty in determining rainfall parameters, such as the probabilities of occurrence and mean rainfall, for weak and strong rain cells. Sometimes these weak and strong rain cells are referred to as *debris* and *cell*, respectively. The model was verified for both the satellite and terrestrial links.

#### 3.2.2. Ghiani Model

This model [73] is based on a PCF-correction-based model for terrestrial links. It can be modeled by simulation with Equation (26) and analyzed with Equation (27):

(26) $$A=\int_{L}\gamma_{R}\left(l\right)dl=\int_{L}kR{\left(l\right)}^{\alpha }dl.$$

(1) Calculate

(27) $$A=k{R_{TX}}^{\alpha }LPF.$$

(2) Calculating the PCF: $PCF=A/k{R_{TX}}^{\alpha }L$ for the number of rain maps generated by the MultiEXCELL model. This results in the following expression:

(28) $$PF_{av}=a\left(f,L\right)e^{-b(f,L)R}+c\left(f,L\right),$$

where the symbols *a*, *b*, and *c* are taken from the regression coefficients. These three coefficients depend on the values of frequency and path length.

(3) Because the effect of the frequency is negligible

(29) $$A\left(P,L\right)=kR{\left(P\right)}^{\alpha }L\left[a\left(L\right)e^{-b(L)R}+c\left(L\right)\right],$$

where the constants are given by the set of equations in (30),

(30) $$\begin{array}{c} \begin{array}{c} a=-0.8743e^{-0.1111R}+0.9061\\ b=-0.0931e^{-0.0183R}+0.1002\\ c=-0.6613e^{-0.178R}+0.3965. \end{array} \end{array}$$

This model’s drawback is that the RMS of the prediction error against the ITU-R database did not exhibit better performance compared to the ITU-R and Brazilian models. Thus, a better terrestrial link rain database from DBSG3 or Comité Consultatif International des Radiocommunications (CCIR) was required for examination before its final application.

#### 3.2.3. Excell/Capsoni Model

The parameters of this statistical model [74] of the horizontal rain structure can be determined based on the local statistical distribution of the point rainfall intensity. The model was validated using the COST 205, 1985 database. This model consists of several rain cell structures, collectively refereed to as kernels. In such a rain cell, the rainfall rate at a distance *l* from the center is given by:

(31) $$R=R_{peak}e^{-l/l_{0}}.$$

Probability of attenuation equation:

(32) $$P\left(A\right)=\int_{R_{E}}^{\infty }E.[0.5\ln^{2}\left(R_{peak}/R_{E}\right)+r\ln\left(R_{peak}/R_{E}\right)].\;[-P\left(R_{p}{)}^{\prime \prime \prime }\right]d\left(\ln R_{peak}\right)$$

where $r=1/4\pi {\bar{l}}_{0}$.

Rain distribution can be calculated as:

(33) $$P\left(R\right)=P_{0}\ln^{n}\left(\frac{R^{*}}{R}\right).$$

Here, P(R)=0 indicates that the probability of rain is zero, which will be true at the rain cell boundary. A simplified version of the model with the point rain intensity at point (x,y) can be defined as:

(34) $$R\left(x,y\right)=R_{M}e^{-\sqrt{{\left(\frac{x}{l_{x}}\right)}^{2}+{\left(\frac{y}{l_{y}}\right)}^{2}}}$$

along a cell radius:

(35) $$R\left(x,y\right)=R_{M}e^{-\frac{\sqrt{x^{2}+y^{2}}}{l_{0}}}.$$

In the sense of the rain attenuation model, this model does not provide attenuation. However, it facilitates the generation of a synthetic rain rate from which attenuation can be predicted using a suitable prediction model. There are critics that the exponential rain peak is not present [75] in nature, and the model does not differentiate between stratiform and convective rain.

### 3.3. Statistical Models

#### 3.3.1. ITU-R Model

This model [50] is primarily based on a distance factor that relies on the rain rate $R_{0.01}$, frequency, link length, and power-law relationship coefficients of the specific attenuation α (furthermore, it is a function of frequency and polarization). The attenuation and the distance factors can be calculated as:

(36) $$A_{0.01}=k{R_{0.01}}^{\alpha }\,dr$$

(37) $$r=\frac{1}{0.477d^{0.633}R_{0.01}^{0.073\alpha }f^{0.123}-10.579\left(1-e^{-0.024d}\right)}.$$

The attenuation, $A_{p}$, which exceeded for a percentage of time *p* other than 0.01%, was determined by the simplification of the attenuation $A_{0.01}$. This model, validated in Malaysia, showed good agreement with the measured attenuation [76].

#### 3.3.2. Singh Model

This model [77] provides an easy calculation mechanism compared to the ITU-R model. The specific attenuation follows the ITU-R model for the frequency band of 1–100 GHz. After calculating the specific attenuation, the curve fitting technique using the MATLAB software cubic polynomial Equation (38) is approximated for the specific attenuation.

(38) $$A\left(dB/km\;\right)=c_{3}f^{3}+c_{2}f^{2}+c_{1}f+c_{0},$$

where the coefficients $c_{3}$, $c_{2}$, $c_{1}$, $c_{0}$ of Equation (38) for the horizontal polarization are given by:

(39) $$\begin{array}{c} \begin{array}{c} c_{3}h=1.422\times 10^{-9}x^{2}+2.03\times 10^{-7}x-1.21\\ c_{2}h=1.963\times 10^{-7}x^{2}+8.618\times 10^{-7}x+0.0019\\ c_{1}h=2.114\times 10^{-6}x^{2}+0.01x-0.036\\ c_{0}h=3.10\times 10^{-5}x^{2}-0.040x-0.031 \end{array} \end{array}$$

and for the vertical polarization:

(40) $$\begin{array}{c} \begin{array}{c} c_{3}v=-5.520\times 10^{-12}x^{3}+3.36\times 10^{-9}x^{2}-1.21\times 10^{-7}x-6.10\times 10^{-6}\\ c_{2}v=8.10\times 10^{-9}x^{3}-4.552\times 10^{-7}x^{2}-3.03\times 10^{-5}x+0.001\\ c_{1}v=-5.71\times 10^{-9}x^{3}+6\times 10^{-7}x^{2}+8.707\times 10^{-3}x-0.018\\ c_{0}v=-1.073\times 10^{-7}x^{3}+1.068\times 10^{-4}x^{2}-0.0598x+0.0442. \end{array} \end{array}$$

A similar approach-based technique was proposed in [78]. However, it was considered the original power-law relationship rather than the simplified polynomial form in that proposal. The second difference is that the constants *k*, α referring to the Equation (42) depends only on frequency and either vertical or horizontal polarization.

(41) $$A\left(\mathrm{dB}/\mathrm{km}\right)=kR^{\alpha }$$

(42) $$\begin{array}{c} \begin{array}{cc} a_{h}=4.21\times 10^{-5}f^{2.42}, & \;\mathrm{for}\;2.9\;\mathrm{GHz}\leq f\leq 54\;\mathrm{GHz}\\ a_{v}=4.09\times 10^{-20}f^{0.069}, & \;\mathrm{for}\;54\;\mathrm{GHz}\leq f\leq 180\;\mathrm{GHz}\\ b_{h}=1.41f^{-0.0779}, & \mathrm{for}\;8.5\;\mathrm{GHz}\leq f\leq 25\;\mathrm{GHz}\\ b_{v}=2063f^{-0.272}, & \mathrm{for}\;25\;\mathrm{GHz}\leq f\leq 164\;\mathrm{GHz}. \end{array} \end{array}$$

### 3.4. Fade Slope Models

#### 3.4.1. Andrade Model

In the Andrade model [79] the variance of the fade slope is proportional to the attenuation as per Equation (43):

(43) $$f\left(f_{s}|A\right)=\frac{1.38}{\sqrt{k\cdot A}\;{\left[1+\frac{{f_{s}}^{2}}{k\cdot A}\right]}^{6.7}}.$$

The predictor can estimate the next attenuation level $A(t_{i}+t_{p})$ from the current attenuation value $A(t_{i})$ and fade slope:

(44) $$A\left(t_{i}+t_{p}\right)=A\left(t_{i}\right)+f_{s}\;t_{p},$$

where $t_{p}$ is the prediction time, it can be considered that $t_{p}=10$, which corresponds to the minimum prediction time, that is, the sampling time of the experimental data.

#### 3.4.2. Chebil Model

In the Chebil model [16] the variance of the fade slope is proportional to the attenuation as per Equation (45):

(45) $$p\left(\xi |A\right)=\frac{1}{\sigma_{\xi }\sqrt{2\pi }}\exp\left(0.5{\left(\frac{\xi }{\sigma_{\xi }}\right)}^{2}\right),$$

where the $\sigma_{\xi }$ is given by Equation (46)

(46) $$\sigma_{\xi }=0.00012A^{3}-0.003A^{2}+0.027A-0.0016.$$

### 3.5. Optimization-Based Models

#### 3.5.1. Develi Model

This model [80] is based on the Differential evolution approach (DEA) optimization technique and experimentally tested at 97 GHz on terrestrial link in the United Kingdom (UK). The steps of the DEA attenuation model are as follows:

(1) The rate of rainfall and percentage of the time exceedance is related to the rain attenuation by equation:

(47) $$z\left(t\right)=\sum_{k=0}^{K}a_{k}x^{k}\left(t\right)+\sum_{n=1}^{N}b_{n}y^{n}\left(t\right),$$

where $a_{k},b_{n}$ (k=0,1,…,K, n=1,2,…,N) are the model parameters. K+N is the total number of input variables in the model.

(2) The mean absolute error is:

(48) $$E=\frac{1}{M}\sum_{k=1}^{M}\left|m_{k}\left(t\right)-z_{k}\left(t\right)\right|,$$

which can be alternatively represented as:

(49) $$E=\frac{1}{M}\sum_{k=1}^{M}\left|m_{k}\left(t\right)-f\left(x_{k}\left(t\right),y_{k}\left(t\right),a_{0},\ldots ,a_{K},b_{1},\ldots ,b_{N}\right)\right|.$$

The mean absolute error given by this equation is treated as the cost function and used to obtain the optimized error by applying the DEA algorithm.

(3) Mutation:

(50) $$\zeta^{M,i}=\zeta^{n,opt}+P_{mut}\left(\zeta^{n,p_{1}}-\zeta^{n,p_{2}}\right),\;\;\mathrm{for}\;i\neq p_{1}\;\;\;\mathrm{and}\;\;\;\;i\neq p_{2},\;$$

where *n* is the generation index, Pmut is the mutation variable, $p_{1},p_{2}$ and *i* are three arbitrarily chosen individual indexes, and the *M* and opt refer to the *gene pool* and the *optimal entity* in the population, respectively.

#### 3.5.2. Livieratos Model

This model [81] was developed using a DBSG3 database-based on a supervised machine-learning (SML) technique. In this rain attenuation model, the SML technique was blended with a Gaussian process (GP). A rain attenuation algorithm must be trained in a particular area of interest to measure the different interdependencies of the parameters for detecting rain attenuation in a specific region, weather, or carrier frequency.

#### 3.5.3. Pinto Model

This model [82] is based on the actual distance correction mechanism through the distance correction factor (*r*) along with the effective rainfall rate distribution ($R_{\mathrm{eff}}$). It uses the quasi-Newton method in addition to particle swarm optimization (PSO); minimizing the root mean square error (RMSE) is the objective function in both cases.

(51) $$A_{p}=k{\left[a_{1}{R_{p}}^{\left(a_{2}+a_{3}/d\;\right)}\right]}^{\alpha }d\cdot \frac{1}{a_{4}d^{a_{5}}{R_{p}}^{a_{6}}f^{a_{7}}+a_{8}\left(a-e^{a_{9}d}\right)}.$$

The $a_{i}\;\left(i=1,2,\ldots ,9\right)$ coefficients can be calculated using quasi-Newton multiple nonlinear regression (QNMRN) and the Gaussian RMSE (GRMSE) algorithm. These coefficients were further fine-tuned using the PSO technique. The model performance has not been compared with the recently developed model, except for ITU-R P.530-17 [50]. Thus, there is a need for further verification before application, except for the temperate climate and Malaysia rainfall database areas.

## 4. Comparative Study of the Models

In the previous section, we have highlighted the working principles of 18 rain attenuation models. In this section, a comparative study of these models is presented. Table 5 presents different input parameters and functional values as inputs to predict the selected models’ attenuation. The link range, supported frequency bands, important regional parameters, supported link types, and remarks about whether the model is spatially friendly are presented in Table 6. Table 5 shows that most terrestrial models have implemented the effective path length technique. The main reason for using path reduction for the long terrestrial link is that several rain cells affect the terrestrial link during rain. As discussed in Section 2.3, the necessity to correct the distance between the transmitter and receiver to estimate the rain attenuation and a few other techniques of determining the effective path are tabulated in Table 3. The suitability of these well-known models to the fitting application area in the climatic region and whether a standard dataset validates these models are tabulated in Table 7. Finally, in Table 8, all the significant contributions, special features, and limitations of the models are presented.

## 5. Model Evaluation Techniques

After developing the rain attenuation model, it should be tested to validate its applicability. There are a few well-known techniques specified by the ITU-R guidelines [83], to test a model’s performance. A different version of the implementation of this guideline is available in the literature. The Equation (52) was used to measure the performance of newly developed model [32,73,84]. The details of the symbols description in Equation (52) are given in [84]. The logarithm value of measured attenuation divided by the predicted attenuation was used as the model evaluation criteria in [71]. The *coefficient of determination* or an *input-to-output correlation function* to determine the performance of the model [82]. The Equation (54) represents the *coefficient of the determination* function. The coefficient of determination, denoted by *R*, is the quotient of the explained variation to the total variation (total sum of squares (TSS)) in a model of simple or multiple linear regression [85]. The RMSE, and the RMS function (Equation (57)) is also used to validate the performance of the rain attenuation model [45,82]. The *goodness-of-fit* function, which in general, measures how well do the observed data correspond to the fitted (assumed) model Equation (55) was also used in [9]. This model is also referred to as the *Pearson goodness-of-fit* statistic function as per given in Equation (56). Furthermore, some researchers used $X^{2}$ (Equation (56)) to find the accuracy of the model [74].

(52) $$V_{i}=\left\{\begin{array}{cc} 100\cdot {\left(\frac{A_{m,i}}{10}\right)}^{0.2}\cdot \ln\left(\frac{A_{p,i}}{A_{m,i}}\right), & 0<A_{m,i}<10\\ 100\cdot \ln\left(\frac{A_{p,i}}{A_{m,i}}\right), & A_{m,i}\geq 10 \end{array}\right.\;$$

(53) $$V_{i}=\ln\left(\frac{A_{measured}}{A_{model-specified}}\right)$$

(54) $$R^{2}=\frac{\mathrm{Explained}\;\mathrm{variation}}{\mathrm{Total}\;\mathrm{variation}}$$

(55) $$\epsilon {\left(P\right)}_{T}=\frac{A_{\%p,\;p}-A_{\%p,\;m}}{A_{\%p,\;m}}\times 100\left[\%\right]$$

(56) $$X^{2}=\sum_{j}\frac{{\left(O_{j}-E_{j}\right)}^{2}}{E_{j}}$$

(57) $$D_{ei}=\sqrt{\mu_{ei}^{2}+\sigma_{ei}^{2}},$$

where $V_{i}$ is the test variable, $A_{\%p,\;m}$ is the predicted attenuation, $A_{m,i}$ is the measured attenuation, $A_{m}\left(dB\right)$ measured attenuation, and $A_{p}\left(dB\right)$ predicted attenuation. The definition of $E_{i}$ and $\sigma_{ei}^{2}$ are given by the equation the equations below.

(58) $$\begin{array}{c} E_{i}=\frac{A_{p_{i}}-A_{m_{i}}}{A_{m_{i}}}\times 100\left(i=1\;\mathrm{to}\;N\right) \end{array}$$

(59) $$\begin{array}{c} \sigma_{ei}^{2}=\frac{1}{N}\sum_{i=1}^{N}e_{i}^{2}-{\left(\mu_{ei}\right)}^{2}. \end{array}$$

**Table 7:** Terrestrial rain attenuation models’ few performance metrics.

Ref.	Suited Area	Validation	Database	mm-wave	Significance of Parameter
[45]	Global (argued)	Validated	Validation database: Congo, Japan, US and Europe, and Malaysia	Highest 75 GHz has been tested	Rain rate statistics related to low-range time percentages and those governed by high-range time percentages may lead to attenuation prediction errors.
[52]	Malaysia	Validated with diverse weather condition’s short-link database	Short-link, rain rate exceeded 0.01% database in Japan, Korea, Spain, New Mexico, and Prague	Yes	Similar to ITU-R
[66]	Unavailable	It needs to validate either through DBSG3 database or experimental database.	No	Yes	Yes
[67]	Temperate region	tested at Paris, Stockholm, Dijon (France), and Kjeller (Norway)	No	Yes	Yes, but there exists few dimensionless coefficients.
[68]		It showed consistency in accuracy for terrestrial and slant links (has not been compared to real measured databases).	ITU-R database	Yes	Yes
[69]	Global	Yes	ITU-R database	Yes	Unavailable
[70]	Malaysia	Yes	Experimental databases	No	Yes
[71]	Global	Yes	CCIR, USA rain databases [86]	Yes	Yes
[72]	Temperate region	It is applicable for slant and terrestrial links	Terrestrial link: 35 path of various countries and slant link: validated through CCIR database	Yes	Yes
[73]	Temperate region	Mean and RMS error prediction based-on the DBSG3 database does not show enhanced accuracy compared to ITU-R model	DBSG3 database	Yes	Yes
[74]	Italy		COST 205, 1985 database	Yes	Yes
[50]	Global	Validated in Malaysia (good agreement with measured attenuation [76])	Experimental database	Yes	Yes
[77]	It is similar to ITU-R model [50].	Validated with the DBSG3 database	Unavailable	Yes	Yes
[79]	Tropical region	Yes	Experimental database	No	Yes
[16]	Malaysia	Yes	Experimental database	Maximum 38 GHz	Yes
[80]	Southern UK	The results showed that the DE based model out performs compared to ITU-R, and ANN-based model.	Experimental database (Southern UK)	Yes (tested: 97 GHz)	The coefficients $\left\{a_{1},\ldots ,a_{5}\right\}$, and $\left\{b_{1},\ldots ,b_{4}\right\}$ have no physical significance.
[81]	Global	Validated in Stockholm, Chibolton, and Tokyo	ITU-R	Yes	Yes
[82]	Global (argued)	The validation results in Malaysia showed least RMSE compared to ITU-R P.530-17 [50], and Crane model [71].	Validated with ITU-R, and Malaysian database	Yes	Yes

**Table 8:** Terrestrial rain attenuation models’ constraints, major contribution, drawbacks, and special feature (if there exists).

Ref.	Constraints	Contribution	Drawbacks	Special Feature (If Any)
[45]	To predict the attenuation, it needs a special rain rate $R_{001}$ (mm/h) that exceeded for 0.01% of time	It proposes effective path length with new functional parameter ζ(L).	It substantially overestimates the measured link attenuation at higher rain rates.	It is suitable for the prediction of the cumulative attenuation.
[52]	The actual reasoning of addressing the high prediction error at a short-link through distance modification factor is not justified.	It gives a solution for ITU-R model [50] for short-link.	The case f≤40 GHz, d<1 km has not been verified.	It was verified through different short-links experimental databases around the globe.
[66]	The model can predict rain limited to 10 km × 10 km, and it has not been tested on a real network environment.	It provides a mechanism to simulate and measure radio link’s throughput. It mathematically calculates the rainfall intensity in the center and in the outer region of the rain structure.	A single rain structure is limited in size.	It facilitates to simulate dynamics behavior of rain owing to link capacity changes by rain attenuation and traffic re-routing.
[67]	Maximum frequency support is 11 GHz.	It proposes a path reduction coefficient as a function of path length and rain rates.	The path reduction coefficient depends on more than 5 km distance while [45] model describes this limit as 7 km.	It proposes to use 1-min rainfall rate using the original Lin’s model [42].
[68]	It has not been tested with real measured attenuation database	It calculates effective path length in a common technique both for terrestrial and slant links, although the vertical aspects differ from the horizontal structure of rain cell.	It is not verified with a real measured attenuation database.	It has tried to unify the path length correction factor.
[69]	It shows good performance for the low percentage of rain rate	It introduced the effective rainfall rate concept.	The tropical climatic case was not tested.	It is applicable for both terrestrial and slant links.
[70]	It is exclusively applicable for the tropical region.	It defined the rate of change of attenuation concerning rain rate.	It needs smaller time percentages in the range 0.005≤%p≤0.001 as the input parameter.	ITU-R model [50] still showed less mean error compared to it.
[71]	It can predicted maximum 30 dB attenuation owing to rain.	It uses rain’s geophysical statistics and rain structure to predict the attenuation of terrestrial and slant links.	It comparatively complicated procedure to calculate attenuation.	It is considered as one of the critical models in practice.
[72]	It is difficult to determine the probabilities of occurrence and mean rainfalls at the center and at the boundary of a rain cell.	It includes the non-uniform heavy and light rain region concept in the signals propagation path.	It is computationally complex: it needs almost ten equations to solve.	It includes the joint statistics required for space diversity system design.
[73]	It needs an additional terrestrial link rain database before final deployment.	It showed to fit spatial rain behavior through matching synthetic rain map and electromagnetic wave.	It showed good accuracy compared to the ITU-R model, but the performance did not exceed the Brazilian and ITU-R models.	The spatial variability of precipitation along terrestrial links is achieved through the synthetic rain cell simulation technique.
[74]	It considers attenuation to be zero below 5 mm/h, especially beyond 20 GHz, which is not justified well.	It integrates rain attenuation, site diversity gain interference by scattering factors.	It needs deployment location’s rain height, which may not be available accurately.	It gives site diversity gain and interference by rain scattering.
[50]	It is not verified well in heavy rainy tropical regions.	It uses horizontal reduction and vertical adjustment factors to predict attenuation.	The path length reduction is inappropriate for a short-range link [46].	It uses 2 parameters called $L_{1}$ and $L_{2}$ of the connection between antennas and RX entrance points (dB).
[77]	The cubic polynomial coefficients were not validated using a separate testing dataset.	It facilities to compute tedious task of computing the *k* and α in ITU-R model.	The applicable climate regions are not defined well.	
[79]	A detailed weather condition is not mentioned in the experimental campaign dataset.	It is one of the most pioneer work that contributes fade slope model for the terrestrial link.	To remove other noise in the fade slope model, a pre-processing stage is normally used; however, no prepossessing was used in it.	It can predict attenuation before 10-s.
[16]	It is a major contribution for fade slope model for terrestrial link.		It did not produce a good fit for an attenuation level of 1 dB.	It showed good performance in the *goodness-of-fit* test.
[80]	For different climatic zones, it needs to determine the different coefficient of rain and percentage of the time.	It showed excellent agreement with the measured values of rain attenuation.	It needs to calculate the coefficients (Taylor’s series fashioned) of rain and % of the time.	The DE optimization algorithm was used as an optimization tool.
[81]	The climatic regions or frequency zones can be facilitated, employing the proper training data set that can convey the experimental information, but it may be difficult to attain.	It is one of the pioneer ML-based rain attenuation models; it does not have geographical limitations.	The tropical behavior of attenuation is not tested yet using this model.	It must train the algorithm with the unique data set in rare climatic conditions.
[82]	The performance was not compared with most latest ITU-R model [87].	It attempted to overcame the main limitation of the original ITU-R model (single value of the rainfall rate cumulative distribution).	The performance was compared with a suspended version ITU-R model.	The parameter adjustment factor was corrected by QNMNR (quasi-Newton multiple nonlinear regression) followed by particle swarm optimization (PSO) technique.

**Table 9:** ITU-R model is ineffective for short distance.

Location [Ref.]	Duration	Link Details	Concentration of Study
Korea [9]	3 years	d:100 m, f:38/75GHz, pol: V	Proposed a new regression-based technique to attenuation prediction at 75 GHz.
Malaysia [52]	1 year	d:300 m, f:26/38 GHz	Find the discrepancy of measured and predicted attenuation through modifying effective distance.
Italy [88]	4 months	d:325 m, f:73/83 GHz	It was a feasibility study of existing model’s prediction capability at *E*-band with short-distance.
UK [89]	1 year	d:35 m, f:25.84/77.52 GHz, pol: V	The wet-antenna effect and impact of rain on the building to building fixed short-range microwave link were analyzed.
Korea [90]	1 year	d:500 m, f:73/83GHz	It was found inconsistency between measured and ITU-R predicted attenuation. So, authors concluded that the ITU-R model is not suitable for a rain rate above 100 mm/h in Korea. However, their outcome contains no information regarding the distance correction factor.
New Mexico [91]	3 months	d:560 m, f:84 GHz, pol: V/H	The experiments were conducted under idealistic condition to avoid other environmental disturbances.
Japan [92]	10 months	d:400 m, f:120 GHz, pol:V	The results show agreement between the measured attenuation and the ITU-R model for the maximum rain rate of 60 mm/h.
Czech Rep. [93]	5 years	d:850 m, f:58 GHz, pol:V	The outcome shows that ITU-R model underestimates the attenuation for both of average yearly or worst-month statics basis.
Albuquerque, NM, USA [94]	May–October (2016–2017)	d:1.7 km, f:72/84 GHz	The findings show that the ITU-R model P.838 model [30] overestimates attenuation and proposed 2 new techniques to calculate specific attenuation with rain rate greater than 40 mm/h.

## 6. Current Research Scope And Challenges

### 6.1. Use of Learning Techniques

In recent technological developments, artificial intelligence (AI) has played an essential role in finding optimized and practical solutions to techniques. Many AI sub-domain techniques and applications have been reported in [12,95,96]. The field of AI has been vast and has existed for many decades. AI can be described as a set that includes both ML and DL. Some significant fields include weather and climate, security, science, policy, mining, medical science, marketing, manufacturing, management, insurance, finance, environmental, engineering, energy, education, and agriculture. Similarly, the AI technique can predict the amount of attenuation and fading margin for a satellite or terrestrial communication radio link. In [97], an AI-based model was developed to predict rain in southern Taiwan by applying the AI technique with data obtained from another region in Taiwan. The prediction was accurate, with minimum error. In the future, this prediction can be used to predict rain attenuation instead of rain rate. Different models consider a few of the available sets of parameters for natural path length correction, contributing to some errors. An effective AI-based path length reduction technique can be applied to obtain an optimum path length; using such a model, and it may be possible to obtain the most accurate path length correction factor.

### 6.2. Need to Access Rain Data Regularly

Because of climate change, the world’s weather conditions, and hence rain event behaviors, are changing [98,99,100]. On account of this, the model will require periodic testing against measured and estimated attenuation to ensure its radio link availability at the desired level. If there is an intolerable difference between the measured and estimated attenuation, the model parameters will require modification. However, if the model estimates attenuation by analyzing a rain database, it may be necessary to update the database with a recent rain rate.

### 6.3. Adoption of Enhanced Synthetic Storm Technique (ESST)

An enhanced synthetic storm mechanism is proposed in [32]. The ESST receives an input report regarding storm velocity and rain height. These parameters can be extracted from other sources with the discriminating procedure of stratiform and convective rain events. Furthermore, depending on the type of event, the model can produce various rain heights. Table 1 presents SST-based few other techniques for rain attenuation time series.

The model can be applied to the earth-space link at 30–300 GHz to calculate the time series attenuation. The experimental results show that the ESST closely aligns with the predicted results. Many models reported in this paper are based on the SST technique, for example, the physical-mathematical models, the global synthetic storm technique (GSST) model, and the artificial neural network-based model. It is expected that the implementation of the ESST technique may yield more accurate predicted values of rain attenuation.

### 6.4. Rain Attenuation Research for 5G and Beyond Network

The effects of rain on the short-distance *E*-band (probable frequency band that can be used in 5G and beyond networks) is highly unpredictable by existing models. The preliminary outcome in [88] shows that the existing general use of the ITU-R model does not fit well to predict rain attenuation for short-ranges <2 km path length.

A continuous 43-month long campaigned experimental data for link length d=2.3 km on attenuation of 72.56 GHz at Budapest with the Telenor Hungary network is reported in [101]. In this work, the authors showed that the ITU-R model predicted higher attenuation than the measured attenuation, especially in the 33–45 dB attenuation range. They proposed a formula to predict attenuation using the following equation:

(60) $$p_{A}=a\cdot \exp\left(-L_{e}b\cdot A\right),$$

where a=0.2991, b=0.1281, and $L_{e}$ is the effective path length.

Article [52] shows experimented results on the terrestrial link at a frequency of 26 GHz and 38 GHz, where the link distance was 300 m between the transmitter and receiver at UTM, Malaysia. The link availability of the 26 GHz and 38 GHz experimental systems was 98.6% and 99.5%, respectively. The visualization of the collected data shows that the well-used ITU-R model [50] does not fit well for distance d<1 km [9,52,88,89,90,91,92]. Table 9 presents some of recent measured attenuation studies that found the drawbacks of the ITU-R model for short-distance links at 26, 38, 58, 72, 73, 75, 77.52, 84 and 120 GHz frequencies.

## 7. Conclusions

This study has conducted the most recent and well-known comprehensive survey of the rain attenuation prediction models for the terrestrial link. The existing models have been classified as physical, statistical, empirical, optimization-based, or fade slope models based on the model development and formulation basis. They have been reviewed concerning innovative concepts, input parameters, advantages, and disadvantages. A careful comparison of several terrestrial link’s rain attenuation model has been introduced and reviewed. According to this survey, no sole prediction model can be regarded as a comprehensive model to satisfy all specifications for diverse infrastructure setup related parameters, geographic locations, or even climatic variations over time. The open research challenges have been addressed to research further the prediction model for the 5G and beyond network’s densely formed microwave and millimeter-wave links. It should be remembered that the rain attenuation prediction model for millimeter-wave applications can also be more effectively developed as most of the currently developed model predictions are yet inaccurate. We believe that this survey will inspire researchers to develop an accurate terrestrial link’s rain attenuation prediction model either at the regional or global level. The comparative study can help people who work in terrestrial link design, link budget planning, and radio wave propagation management areas.

## Figures and Tables

**Table 5:** Related input parameters/functions of terrestrial rain attenuation models.

		Parameters											
Models	Ref.	➀ Path	➁ Frequency	➂ Rain Rate	➃ Time Series (Attenuation)	➄ Rain Rate Exceeded	➅ Rain Height	➆ Polarization	➇ Humidity	➈ Latitude, Longitude	➉ Effective Path	⑪ CCDF	⑫ Effective Rainfall
Empirical	[45]	✓	✓	✓		✓					✓		
	[52]	✓	✓			✓					✓		
	[66]		✓	✓				✓					
	[67]	✓	✓	✓				✓					
	[68]	✓	✓	✓		✓		✓			✓		
	[69]	✓	✓	✓	✓	✓					✓		✓
	[70]	✓	✓	✓		✓		✓			✓		
	[71]	✓	✓	✓		✓			✓	✓			
Physical	[72]	✓	✓	✓			✓	✓					
	[73]	✓	✓	✓				✓			✓	✓P(R)	
	[74]	✓	✓	✓	✓		✓	✓					
Statistical	[50]	✓	✓	✓				✓		✓	✓		
	[77]	✓	✓	✓				✓		✓	✓		
>Fade slope	[79]	✓	✓	✓	✓			✓				✓	
	[16]	✓	✓	✓	✓			✓				✓	
>Optimization-based	[80]			✓		✓					✓		
	[81]	✓	✓	✓		✓		✓			✓		
	[82]	✓	✓	✓		✓		✓					✓

**Table 6:** Properties of rain attenuation models.

		Parameters				
Models	Ref.	➀ Link Range	➁ Frequency Range	➂ Regional Parameters	➃ Supported Link Type	➄ Spatially Friendly
Empirical	[45]	58 km	7–38 GHz	rain rate	terrestrial	unavailable
	[52]	300 m	26–75 GHz ${}^{⧫}$	rain rate	terrestrial	short-link; spatial may not be important
	[66]	2 km - —	unavailable ${}^{▴}$		terrestrial, satellite	10 km
	[67]	—	12 GHz	rain rate	terrestrial	—
	[68]	0.5–58 km	7–137 GHz	rain rate	terrestrial, satellite	✓
	[69]	unavailable	1–100 GHz	rain rate	terrestrial, satellite	✓
	[70]	3.58–21.7 km	14.8–38 GHz	rain rate	terrestrial	✓(knowledge of long-term rainfall rate).
	[71]	10–60 km	11–36.5 GHz	rain rate	terrestrial, satellite	within 22.5 km spatial independence.
Physical	[72]	1.3–58 km	1–100 GHz	cell: rain and debris	terrestrial, satellite	spatial correlation function.
	[73]	—	—	rain rate	terrestrial	✓
	[74]	—	10–20 GHz	rain rate	terrestrial	✓
Statistical	[50]	2 ${}^{♣}$–60 km	1–100 GHz	rain rate	terrestrial, satellite	up to 110 km
	[77]	2 ${}^{♠}$–60 km	1–100 GHz	rain rate	terrestrial, satellite	unavailable
Fade slope	[79]	12.8–43 km	14.5 GHz	rain rate	terrestrial	unavailable
	[16]	300 m	38 GHz	rain rate	terrestrial	unavailable
Optimization-based	[80]	6.526 km	97 GHz	rain rate	terrestrial	unavailable
	[81]	0.5–58 km	7–137 GHz	rain rate	terrestrial	unavailable
	[82]	0.5–58 km	7–137 GHz	rain rate	terrestrial	unavailable

⧫: Tested, ▴: 80 GHz corresponding rain attenuation with vertical polarization is mentioned, ♣,♠: Recent study showed that ITU-R model overestimates attenuation within short-link about within 2 km, —: unavailable.

## Data Availability

Not applicable.

## Abbreviations

The following abbreviations are used in this manuscript:

A	Rain attenuation
AI	Artificial intelligence
ANN	Artifitial neural network
CCDF	Complementary cumulative distribution function
CDF	Cumulative distribution function
CCIR	Comité Consultatif International des Radiocommunications
CHIRPS	Climate Hazards Group InfraRed Precipitation with Stations
CML	Commertial microwave link
DBSG3	Study group 3 databanks
DE	Differential evolution
DEA	Differential evolution approach
DL	Deep learning
ECMWF	European Centre for Medium-Range Weather Forecasts
EPL	Effective path length
ERA	ECMWF re-analysis
ESST	Enhanced synthetic storm technique
EXCELL	EXponential CELL
FMT	Fade mitigation technique
GP	Gaussian process
GPR	Gaussian process regression
GRMSE	Gaussian RMSE
GSST	Global synthetic storm technique
IDW	Inverse distance weighting
ITU-R	International Telecommunication Union- Radio-communication sector
LOS	Line of sight
LPF	Path length factor
ML	Machine learning
mm-wave	Millimeter-wave
NOAA	National Oceanic and Atmospheric Administration
NRT	Near real-time
NWP	Numerical weather prediction
PCF	Path length coefficient factor
PDF	Probability density function
POI	Point of inflexion (a point after which the rain rate increases abruptly)
PSO	Particle swarm optimization
QNMRN	Quasi-Newton multiple nonlinear regression
RF	Radio frequency
RMS	Root mean square
RMSE	Root mean square error
SML	Supervised machine learning
SST	Synthetic storm technique
STD	Standard deviation
T-C	Two-component
TPW	Total precipitable water
TSS	Total sum of square
UK	United Kingdom
UTM	Universiti Teknologi Malaysia
**Meanings of Used Symbols**	
$\beta_{a},v_{a}$	Gamma distribution parameters
$\Delta x_{0}$	Shift due to the presence of layer B (of A, B layer rain structure)
γ	Specific attenuation
*ℑ*	Fourier transforms
${ℑ}^{-1}$	Inverse Fourier transforms
$\gamma_{R}\left(t\right)$	Specific attenuation
$A_{p}$	Rain attenuation exceeded at p% of time
$c_{1},\ldots ,c_{4}$	Constants that depend on the geographical area
$h_{R}$	Rain height (km)
$L_{e}$	Effective path length
$R_{0.01}$	denotes rain rate at 0.01% of time
$R_{1-min}$	1 min rain rate
$R_{p}$/R(P)	Rain rate exceedance of the p% of the time.
${R_{i}}^{S-B}$	Rain rate due to Salonen-Poiares Baptista method
$A(R_{1},L)$	Rain attenuation
$a_{0}$	Initial condition of rain attenuation (between 0 to 0.5 dB)
*d*	True path length
$d_{0}$	Equivalent cell diameter
$d_{AG}$	Dynamic parameter β of the Maseng-Bakken model, denotes rate of change
$d_{eff}$	Effective path length
$I_{f\gamma }$	Increment factor, where $I_{f\gamma }=\frac{\gamma_{eff}}{\gamma_{th}}$ for d<1 km
$K_{dp}$	Specific differential phase
$L_{A},L_{B}$	Radio path lengths in two layer structure of rain
$M_{z}\left(d\right)$	Correlation of a Gaussian random process
$N_{e}$	Number of effective cells
P(R)	Probability as a function of rainfall rate
$R_{effT}$	Effective rain rate for terrestrial links
S(R%p)	Slope of attenuation with respect to rain rate
$T_{s}$	Sampling time
W(t)	Wiener process
$W_{i}$	Weight for the *i*th site
x(t)	Percentage of the time
$x_{0}$	Distance given by advacation velocity of rain (SST model: $x_{0}=v\cdot t$)
y(t)	Rate of rainfall
z(t)	Rain attenuation
$Z_{dr}$	Differential reflectivity
$Z_{h}$	Radar reflectivity
